# Targeted capillary photothrombosis via multiphoton excitation of Rose Bengal

**DOI:** 10.1177/0271678X231151560

**Published:** 2023-01-17

**Authors:** Patrick Delafontaine-Martel, Cong Zhang, Xuecong Lu, Rafat Damseh, Frédéric Lesage, Paul J Marchand

**Affiliations:** 1 Department of Electrical Engineering, Polytechnique Montreal, Montreal, Canada; 2 Research Center, Montreal Heart Institute, Montreal, Canada; 3 DeGroote School of Business – McMaster University, Ontario, Canada; 4 College of Information Technology, United Arab Emirates University, Al Ain, United Arab Emirates; 5 École polytechnique fédérale de Lausanne- EPFL, Lausanne, Switzerland

**Keywords:** Capillary, multiphoton application, OCMA, photothrombosis, Rose Bengal, two-photon microscopy

## Abstract

Microvascular stalling, the process occurring when a capillary temporarily loses perfusion, has gained increasing interest in recent years through its demonstrated presence in various neuropathologies. Studying the impact of such stalls on the surrounding brain tissue is of paramount importance to understand their role in such diseases. Despite efforts trying to study the stalling events, investigations are hampered by their elusiveness and scarcity. In an attempt to alleviate these hurdles, we present here a novel methodology enabling transient occlusions of targeted microvascular segments through multiphoton excitation of Rose Bengal, an established photothrombotic agent. With n = 7 mice C57BL/6 J (5 males and 2 females) and 95 photothrombosis trials, we demonstrate the ability of triggering reversible blockages by illuminating a capillary segment during ∼300 s at 1000 nm, using a standard Ti:Sapphire femtosecond laser. Furthermore, we performed concurrent Optical Coherence Microscopy (OCM) angiography imaging of the microvascular network to highlight the specificity of the targeted occlusion and its duration. Through comparison with a control group, we conclude that blood flow cessation is indeed created by the photothrombotic agent via multiphoton excitation and is temporary, followed by a flow recovery in less than 24 h. Moreover, Immunohistology points toward a stalling mechanism driven by adherence of the neutrophil in the vascular lumen. This observation seems to be promoted by the inflammation locally created via multiphoton activation of Rose Bengal.

## Introduction

Recent studies have highlighted the role of an increase in transitory losses of perfusion in capillaries (so-called capillary stalls) in various pathologies. An increase in the frequency and duration of such events has been observed in Alzheimer's disease,^1^ acute stroke^2^ and following targeted occlusion of a penetrating arteriole.^3^ Using two-photon microscopy, Cruz Hernandez et al. found these stalls to be caused by the temporary adhesion of neutrophils to the capillary wall, ultimately blocking the passage of red blood cells (RBC).^1^

Despite the increasing evidence for the presence of stalls in these neuropathologies, studying their impact on the neighboring tissue is cumbersome, due to their spatial and temporal rarity.^4^ Simulations have highlighted the potential of such flow interruptions in causing hypoxic pockets,^5^ as seen in elderly mice,^6^ however *in vivo* evidence highlighting the causal relationship between both observations is still lacking. Moreover, Schmid et al. pointed out through simulations that vessel topology is a factor determining the local impact of stalls, but validation of these predictions are still to be confirmed in an *in vivo* setting.^7^ Current investigations of capillary stalling rely primarily on very long acquisition times, to maximize the probability of observing these events. Although the aforementioned methodology enables mapping of relatively large microvascular regions, it is not ideally suited to study the physiological impact of capillary flow interruptions on surrounding tissue. Ultimately, such in-depth investigations could be made possible through two main strategies, either by increasing the imaging throughput, or by devising a way to trigger such microvascular perfusion losses. The work outlined here focuses on the latter scheme.

Photothrombotic models offer an interesting methodology to perturb the cerebral microvasculature, wherein single arteries or venules can be selectively occluded by photostimulation following the injection of a photothrombotic agent, like Rose Bengal.^8^ The excitation of photosensitive agents at a specific illumination wavelength will generate reactive oxygen species (ROS), activate platelets and damage the endothelium, ultimately leading to a clotting cascade.

Such models have been used extensively, to study the impact of a single penetrating arteriole occlusion on somatosensory function,^9^ to investigate vascular remodeling post-occlusion^3,10^ and to delineate oxygenation changes caused by such vascular lesions.^11^

An unfortunate shortcoming of this model is the potential secondary occlusions in the surrounding microvasculature caused by the scattering of the laser illumination by tissue.^12^ As the process relies on single-photon absorption of the photosensitive dye, every vessel illuminated by the excitation beam is susceptible to a thrombotic event. The region of the targeted lesion can therefore be larger than the one defined by the light beam due to scattering. Hence, developing a method answering these limitations is of paramount importance to ensure selectivity of the targeted capillary occlusions.

The advent of multiphoton imaging has provided novel tools for studying and manipulating cortical physiology at high resolution in a depth-resolved manner,^13^ enabling imaging and triggering neuronal activity,^14,15^ measuring blood flow velocity^16^ and even tissue oxygenation using phosphorescent dyes.^17^ In contrast to confocal imaging wherein the depth-sectioning is performed by filtering out-of-focus light through a detection pinhole, the multiphoton absorption process naturally confines the excitation axially, limiting significantly any excitation of structures outside of the focal volume. Furthermore, the imaging depth obtainable in multiphoton microscopy is increased compared to its single-photon counterpart, as its illumination is typically in the near-infrared wavelength range and is thus slightly less prone to scattering.

Building on this advantage, Nishimura et al. demonstrated selective occlusions of single vessels in the neocortex by triggering a clotting cascade through non-linear cavitation using ultrafast amplified lasers.^18^ Using their devised methodology, Shih et al. demonstrated the ability to occlude arterioles and capillaries with high selectivity to study the impact of their blockage on cortical function.^9^ Nevertheless, although the proposed method could reliably cause targeted micro-infarcts, its implementation necessitates the addition of high-energy pulsed lasers and the micro-insults often result in irreversible sectioning of the targeted vessel creating micro-bleeds.

In view of these limitations, we propose a novel method for targeted photothrombosis, through multiphoton excitation of a well established photothrombotic agent. The strategy we devised here employs a Ti:Sapphire femtosecond laser, ubiquitous in any neuroscience laboratory comprising a two-photon microscope. We developed a method of selected capillary stroke that could be used to disrupt a vascular architecture and shed light on their contributions to alterations in local flow, oxygenation or local inflammation. Overall, we demonstrate that by stimulating Rose Bengal using femtosecond pulses at 1000 nm for less than 5 minutes, we could create transient targeted microvascular occlusions or reversible blockage, up to a depth of ∼250 µm in the cortex during 4 hours or more.

## Material and methods

### Animal groups

The Animal Research Ethics Committee of the Montreal Heart Institute approved all procedures described here, in accordance with the Canadian Council on Animal Care recommendations and the protocol for this study was accepted under the ID 2020-2496, 2019-32-02. We applied the recommendation of the ARRIVE guidelines during this study. Mice underwent a diet of TEKLAD GlobaL 19% protein extruded rodent diet (Envigo) and were under a light/dark cycle of 12 hours each. Clean drinkable water was at all times available. A total of n = 16 mice were used in this work. We used n = 7 C57BL/6 J mice (5 males and 2 females, 3 to 6 months old) kept in separate cages. Although the study involved a Rose Bengal and a control FitC group, the same mice were used for both groups, but were imaged on different days and in different cortical areas. Two distinct regions were randomly selected on the surface of each mouse’s cranial window, for a control session with the FitC and the Rose Bengal trial (see cranial surgery section for procedure). Several days separated the control and the Rose Bengal trial (minimum of 3 days). The control experiment, involving a tail-vein injection of FitC (50 mg/mL in saline, volume of 7 uL/g body weight, Sigma-aldrich) was performed, followed by selection and excitation of capillaries up to 250 um deep under the pia (see section Multiphoton photothrombosis imaging sessions). Two days later, the mouse underwent an intraperitoneal injection of Rose Bengal (15 mg/ml in saline, volume of 7uL/g body weight, Sigma-aldrich) and photothrombosis insults were induced by performing the excitation protocol in the selected region. These groups were designed to verify that photo-ablation was not the mechanism leading to the observed insults and to assess the variability of insult type (blockage, hemorrhage or extravasation). A subset of 2 mice were monitored before the insult, post insult and 24 hours after. An additional group of n = 3 mice were monitored before the insult, after the insult, 2 hours, 4 hours and 24 hours following the insult to gather data on the length of blockage from this methodology (See supplementary figure 1). An additional 6 mice were used under the same imaging conditions to undergo brain histology and were separated in 2 groups (n = 3 4 h post insult and n = 3 24 h post insults)(see Histology). At all times, P.D-M., P.J.M and F.L. were aware of the mice’s location and experiment days.

### Cranial surgery

To allow imaging of the cortex, a conventional craniotomy was performed on the mice up to ten days before imaging. The craniotomy protocol used in this work was identical as described in Lu et al.^20^ Throughout the surgery, a platform (LabeoTech, Canada) monitored the temperature via a rectal thermometer, respiration rate and heart rate via electrocardiogram. Ketoprofen (5 mg/Kg, Merial, Canada) and buprenorphine (0.05 mg/Kg, Reckitt Benckiser Healthcare, UK) were injected before the surgery for analgesic purpose. Under anesthesia (2% isoflurane in oxygen), the scalp was removed, and the skull was cleaned to remove left-over conjunctive tissue. A small area of the skull was thereafter removed with a microdrill. Dental cement was then used to fix the window onto the exposed area. A titanium bar was glued onto the head to serve as an anchor point to maintain the mouse brain fixed during the imaging sessions. Baytril (5 mg/Kg, Bayer, Germany) was injected after surgery. Doses were repeated 24 h after and supplemental buprenorphine was used if signs of pain were manifested by the animal up to 24 h after the surgery. Annexed to the imaging system, a custom wheel and fixation fork was utilized to maintain the mice’s head fixed with the titanium cranial bar. To reduce stress, since the animals were imaged awake, training through a gradual increase of head-fixation time on the wheel was performed over 4 days from 10 minutes to 45 min (after 3 days of recovery post-surgery).

### Multiphoton photothrombosis imaging sessions

**Figure 1.**
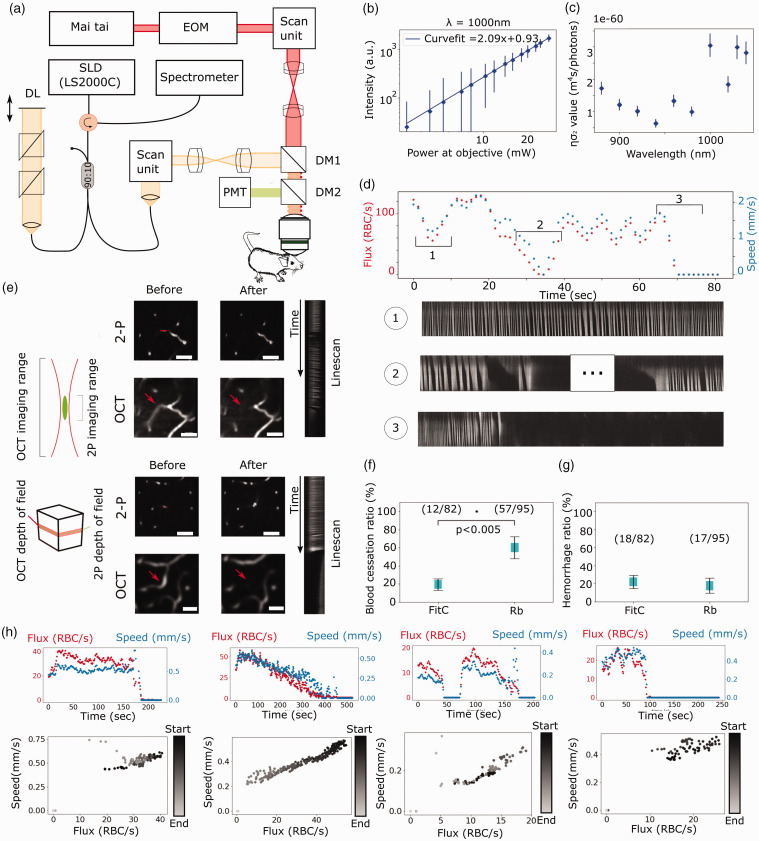
Overall view of the imaging system (a). EOM: Electro-optical modulator, PMT: photo-multiplier tube, DM1 and DM2: Dichroic Mirrors (Longpass), DL: optical Delay Line. Log-log plot of 2 P excitation signal from Rose Bengal dye exhibiting a slope of 2.09 (b) and the cross section of the Rose Bengal (c). Using the cross section data, one can see a peak cross section value at 1000 nm used in this study. Typical demonstration of a blockage session (d).The longer depth of field in OCMA provides more spatial information with an angiogram protocol and the respective blockage events are shown in the line scans (e). Scales bars are 100 um, red arrows show blood flow cessation in targeted vessels and red line is site of Rose Bengal excitation. Reckoning of blockages and hemorrhage incidence rates (f and g). RBC flux and speed during Rose bengal excitation formation sessions with their respective correlation plot (h).

For the imaging procedure, the mice were placed on a head-fixation stage and put under the microscope for a maximum duration of 2 hours (Microscope described in supp. methods, Figure 1(a)).^3,4,19^ Imaging sessions consisted of 2D multiphoton acquisitions to detect potential target capillaries in one of the selected regions at 100 µm of depth and up to 250 µm below the pia. The power of the laser after the 25× objective was around 87 mW at 1000 nm. No additional dyes were used during the imaging sessions since multiphoton excitation of Rose Bengal yields fluorescence signals at such wavelengths. To ensure maximal power at the focal point, we avoided capillaries lying underneath large cortical vascular structures (veins and arteries). After selecting the target capillary, line scans were performed approximately 15 µm along the vessel's axis at 800 Hz to excite the Rose Bengal. The status of the insults was monitored by observing the intensity of the plasma and the passage of RBCs. 2P excitation of Rose Bengal was done until blood flow interruption was observed in the targeted vessel, 2P line scans data were recorded for an additional 15 seconds or more to ensure blood flow cessation. An additional verification scan was done up to 5 minutes after the insult. If both measures confirmed a blocked vessel, we treated the vessel as blocked.

### Histology

We performed histological analysis on the cortical region of interest where up to 15 insults were performed in an area of 1 mm × 1 mm. Animals were anesthetized four hours or 24 hours following the imaging session with 2% isoflurane and received intracardiac perfusion through the apex of the heart with 30-mL phosphate buffered saline (PBS, 0.3 mL/min) followed by 30 mL of 4% paraformaldehyde (PFA) in PBS also at a rate of 0.3 mL/min. The brains were then extracted and placed in a 4% PFA solution during 24 h. Following a dehydration process and an embedding in paraffin, the brain was cut in 6 µm thick slices to undergo histological marking of neutrophils, VCAM-1, platelets (CD41), E-selectin (CD62) and neutrophils (Ly6G) (see supplementary methods).

### Exclusion criterion

**Figure 2.**
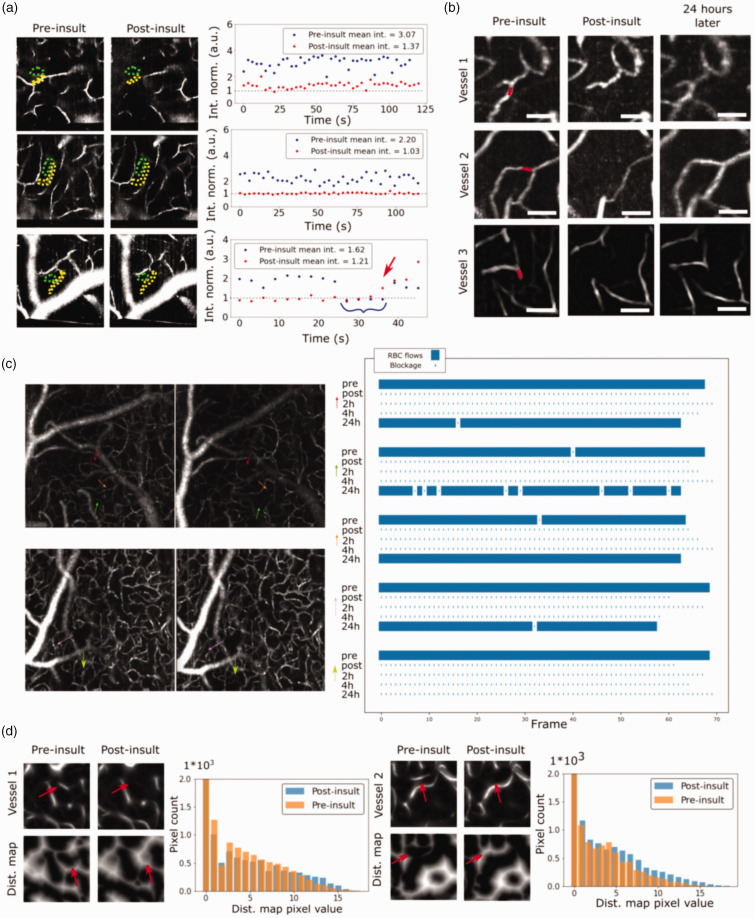
Examples of different blood flow dynamics in different capillaries via OCMA (a). The green region represents the background used for normalization of the signal shown in the yellow region. Three recurrent observations are presented: RBC flux reduction, RBC flux cessation or rapid recovering of the RBC flux. The baseline intensity of 1 is represented by the gray dotted line for visual assessment. Persistence of the insult can be assessed with temporal imaging and 24 hours reevaluation (b). Showing examples of 3 vessels, OCMA intensity vanishing shows the RBC flux cessation in the vessel. The red line shows the 2P line scan excitation localization and the scale bar is 100 um. After 24 hours, the targeted vessel recovers its RBC flux. Targeted vessels seem to Continued.exhibit a total flow blockage up to 4 hours post thrombosis (c). But, the recovered flow may not exhibit the same hemodynamics following the insult. Colored arrows point to the targeted vessel in the angiogram field of view. During an insult, nearest distance from a vessel is lengthened (d). 2 vessel blockages are shown with their impact on the distance histogram and red arrows point to the targeted capillary. Followed by image processing to show the distance from the nearest vessel, higher values in the histogram are promoted, showing potential for impaired nutrient delivery. Peripheral vessels may not be affected by the insults inflicted via the resilience of the vascular system (d). Vessels labelled yellow are peripheral to the targeted red one and the red dotted line is the excitation line scan path (left). Despite changes in the signal, peripheral vessels exhibit OCMA signals even after the insult took place (right).

After cranial surgery, the experiment with a particular mouse was interrupted if signs of pain were to remain past 24 h of recovery. No full 2P excitation line scans were excluded but the criteria for exclusion was bad signal from the 2P line scans that could come from an optical system misalignment, dye injection failure or observation of cortical inflammation, dura growth and RBC speed/flux data arising from erroneous data due to movements artifacts. The first 10 mice were considered for the analysis of the Rose Bengal excitation, insults and correlation (Figures 1 (f) to (h) and 2(a)). Additional criteria for exclusion during analysis were low quality optical coherence microscopy angiography (OCMA, see supp. methods) imaging from which 2 samples were excluded. As for the analysis of blockage dynamics shown in Figure 2(b) and (d), a subset of n = 2 mice across 5 imaging sessions were used with the exclusion of two imaging sessions due to artifacts. An additional experiment monitoring the blockages of the insulted vessels with n = 4 mice was conducted for Figure 2(c). One mouse of this group was excluded due to issues in the integrity of the craniotomy and a single imaging session was performed for each of the 3 other mice. In the immunofluorescence trials, 2 mice of each group had to be excluded since planning of these experiments required practice trials (see supp. methods for more info on analysis^21^^–^^25^).

## Results

### Multiphoton excitation of Rose Bengal for photothrombosis

Prior to inducing insults *in vivo*, we characterized the multiphoton excitation properties of Rose Bengal. We measured the relationship between the input illumination power at 1000 nm and the fluorescence emission of the Rose Bengal at 580 nm. We filled an optical chamber with Rose Bengal and measured the output fluorescence with a photomultiplier tube (PMT) under increasing excitation powers. Figure 1(b) shows the polynomial response of the emitted fluorescence under two photon (2P) excitation. The slope of the fluorescence in a log-log plot has a value of 2.09 suggesting a 2P excitation process. The maximal power output measured at the front focal plane of the microscope objective was ∼87 mW. Secondly, we computed the cross-section of Rose Bengal using the method delineated by Xu et al.,^26^ by varying the laser's emission wavelength from 850 nm to 1040 nm. As can be observed in Figure 1(c), the obtained cross section variations across the laser's spectral range indicate the presence of excitation wavelength candidates. We chose to excite the photosensitive dye at 1000 nm since the power of our laser source was higher compared to 1040 nm. The cross section measured at 1000 nm for Rose Bengal was (3 ± 0.3) × 10^−60^ m^4^s/photons compared to Fluorescein isothiocyanate (FitC) at 1000 nm which is around 8 × 10^−58^ m^4^s/photons representing a 100-fold reduction.^26^ These results highlight the potential for multiphoton fluorescence excitation of Rose Bengal at 1000 nm. We then sought to verify the photothrombotic capabilities of the non-linear excitation of Rose Bengal. We thus performed line scans along the longitudinal axis of capillary blood vessels *in vivo*, enabling both the measurement of hemodynamic parameters (RBC speed and flux) and the activation of Rose Bengal, as shown in Figure 1(d). With the same agent, the highlighted timetrace presents the variability in RBC flux and RBC speed observed during the excitation process in the 2P imaging setup. As is shown in the section of the timetrace labeled “1”, we provide a timetrace representative of the initial RBC flow in the targeted vascular structure. In the time trace labeled “2”, we observed intermittent stalling events during the excitation protocol, followed by a recovery of the initial RBC flux and speed. Lastly, as is shown in the timetrace labeled “3”, we observed a persistent blockage, characterized by an absence of RBC lasting 15 seconds or more. As mentioned above, isolated blood flow cessation events were observed before a total RBC flow cessation. For this reason, we set a minimal time of 15 seconds of continuous blockage absence of RBC to consider the vessel blocked via the Rose Bengal excitation. The example shown in Figure 1(d) and supplementary video 1 also represents a typical blood flow cessation observed with the multiphoton signal from the Rose Bengal fluorescence. In summary, Rose Bengal is a 2P fluorescent dye which can be used as a monitoring tool and photothrombotic agent.

### Insults etiology

As detailed in Nishimura et al, the different flow behaviors or insults seen in line scans and in two-dimensional acquisitions could be explained by distinct mechanisms, such as a blood clot formation, extravasation or explosion of the vessel (bleed). Throughout our targeted photothrombosis experiments, we also observed these three types of vascular insults.

The most common observation consisted in a blood flow interruption causing little or no spill of the imaging dye in the extracellular space, as shown in Figure 1(e). Alternatively, in some cases the excitation process could lead to a rupture of the vascular wall resulting in a spill of the imaging dye in the neuropil. Lastly, we also observed blood flow cessation events followed closely by an inflation of the vessel until rupture of the vascular wall. The two latter observations were labeled as hemorrhages since these insults were not in the scope of the study.

To discard the possibility that the thrombi made in this experiment were induced by laser damage, we compared the ratio of occlusions between a control group injected with FitC and another with Rose Bengal at similar power levels. The results of these experiments are shown in the Figure 1(f) and (g). We show the total amount of trials and the respective occurrences of blood flow interruption for both blockage and hemorrhages (Figure 1( f) and (g) respectively). As is highlighted in Figure 1(f), the ratio of blockage formation over 95 trials (60%) is almost five-time higher for the Rose Bengal excitation than with FitC (12 blockages in 82 trials (14%)). This result strongly suggests that Rose Bengal played a role in the blockage mechanism. Conversely, the hemorrhage ratio was similar between both groups, as is shown in Figure 1(g).

We additionally investigated the average excitation time necessary for blockages to occur, and obtained 295 ± 270 seconds, 290 ± 173 s and 317 ± 226 s for depths of 100 µm, 150 µm and 200 µm below the pia respectively.

To assess the performance of the Rose Bengal multiphoton excitation, we devised a proportion z test defining the blood flow interruption without hemorrhage as a binary positive value and the rest of observations as a null value. This test gave a p-value of 10^−9^ showing a quantitative rejection of the null hypothesis between the FitC injection group and the Rose Bengal injection group. The null hypothesis cannot be discarded for the hemorrhage ratio, as both groups exhibit a similar rate of this kind of vascular insult.

### Speed-flux correlation conservation

With the photothrombotic protocol in place, we sought to investigate the hemodynamic changes occurring during excitation. Figure 1(h) presents the RBC flux and speed timetraces for four vessels throughout the photo-excitation. RBC speed profiles from the 2P line scans were analyzed and artifacts induced by sample motion were dismissed in the computing of speed and flux (see methods).^21^ As can be seen in the individual panels, despite some erroneous data in the speed profile, RBC hemodynamics correlate until the blockage is observed. This correlation is highlighted in the scatter plots placed below each individual hemodynamic timetrace. Interestingly, although the RBC flux and speed are both reduced during the excitation, their linear dependency remains even for low values. During the excitation of Rose Bengal, no clear trend of RBC flux and speed reduction is apparent from the excitation initiation (Figure 1(h), supplementary figure 2), except for some cases such as the second timetrace where a clear descending slope is observed.

### Multimodal imaging of capillary events

As demonstrated previously, through multiphoton excitation, Rose Bengal can be used both as an imaging dye and as a photothrombotic agent. Although this behavior allows concurrent hemodynamic monitoring and photothrombotic stimulation, as was shown above, it also restricts the imaging conditions. In order to assess the perfusion status of the surrounding vasculature without triggering the thrombotic agent, we therefore imaged the angioarchitecture using the OCMA modality of our imaging platform. As OCM is a label-free imaging technique operating at 1300 nm, it ensured no interference with the multiphoton detection and no activation of the dye. Furthermore, the OCMA path having its own independent scan unit enabled imaging a different region than the one interrogated by the multiphoton path. Figure 1(e) highlights two examples of blockage observed in 2P and OCMA. The red line in the 2P image shows the line over which the line scan protocol was performed. As can be observed in these 2P angiograms, the 2P's optical sectioning is extremely narrow and thus fails to provide a confirmation of blood flow cessation, as an axial displacement artifact could render the same effect. However, OCMA is an imaging modality which has a greater depth of field and is able to provide so-called perfusion maps wherein static structures and dynamic ones can be differentiated by high pass temporal filtering.^26^ If a vessel is irrigated, it will appear as a bright feature in the angiogram. Conversely, if the vessel is blocked, no RBCs will flow through it and the structure will be indistinguishable from the static background. As seen in the corresponding OCMA acquisition in Figure 1(e), one can distinguish the similarities of observable structures during the irrigation of the vessels between the two modalities. Then, the blockage of the targeted capillary is assessed through the vanishing of the vessel in OCMA.

### Insults dynamics

To investigate the temporal characteristics of the insult by imaging before and after multiphoton thrombosis, we used OCMA acquisitions as a means of qualitatively characterizing the hemodynamic status of targeted capillaries (i.e. the irrigation of the vessel and its surrounding) similarly to Srinivasan et al.^26^ Figure 2(a) shows three different acquisitions with this imaging scheme. To assess the perfusion state inside the targeted vessel, the intensity of the vascular structure in the perfusion map is normalized to the local background. The right panels present timetraces of the vessel's brightness segmented by a yellow region normalized to the background in the respective green region. In these plots, OCMA timetraces manifest either a higher intensity compared to the baseline timetrace (erythrocytes are passing through the vessel), similar intensity as the background (representing a total blockage) or both (corresponding to intermittent stalling events). The first and second acquisition show a significant change in the irrigation of the targeted vessels post-insult. These results corroborate the RBC flow cessation in the 2P timetraces. The third plot of Figure 2(a), shows a pre-insult stalling event highlighted with a blue curly brace. This vessel then exhibits a rapid unblocking after the insult (around 35 seconds as pointed out by a red arrow). The vessel, therefore, experienced a flow interruption before and during the 2P line scan excitation, but regained irrigation once the excitation was halted. These observations corroborate the targeted multiphoton thrombosis done via the 2P optical path and enable the monitoring of insulted vessels without undesired activation of the thrombi agent.

### Insult duration

The rapid recovery post-insult observed in the Figure 2(a) raises questions regarding the duration of the photo-induced capillary flow cessation. In order to characterize the durability of the insult caused by the presented protocol, we thus tracked and imaged targeted vessels using the OCMA imaging path before and immediately after the blood flow cessation, 2 hours, 4 hours and 24 hours later. Figures 2(b) and (c) show the OCMA resulting from this sequence of acquisitions. Immediately following the protocol, the downstream vessel's intensity captured in OCMA shows a blood flow cessation (the vessel is indistinguishable from the background).

The imaging sessions performed 2 and 4 hours post thrombosis highlight a persistent blockage of the targeted vessel as shown in the stallograms. However, 24 hours after the insult, most tracked vessels appear once again in OCMA (n = 15 observations) but don't necessarily exhibit more events of stallings, demonstrating that the protocol produces under 24 hours blockages rather than creating vessels exhibiting stalling events.

### Targeted photo-occlusions lead to larger distances between vessels

During the occlusion, nutrient delivery may be impaired in the vicinity of the targeted vessel. In an attempt to shed light on the effect of the blood flow cessations induced by our protocol, we computed the distances from the nearest vessel before and after targeted photo-thrombosis. Once again, we selected OCMA as a means of obtaining a 3D angiogram to prevent any undesired photo-excitation of the vascular arbor during imaging. The 3D OCM angiograms were then processed through various steps (see methods) to obtain an estimation of the distance between each point in tissue to its nearest vessel, so called *distance map*. Figure 2(d) presents OCM angiograms obtained before and after photo-thrombosis and their corresponding distance maps, for two distinct vessels. A distinct difference can be observed in the distance maps of the angiograms before and after photo-occlusion, as is pointed out by the arrows in Figure 2(d). We attempted to quantify these observations by comparing the histograms of the distance maps before and after the insult. Care was taken to precisely superimpose both angiograms prior to obtaining the histogram. A shift in the histogram can be observed in Figure 2(d), confirming our initial claim that the photo-thrombosis leads to slightly longer distances between vessels. These longer gaps could potentially lead to nutrients delivery impairments and may cause harm to cellular bodies in the neuropil where longer distances from a vessel are observed.

### Histological markers

**Figure 3.**
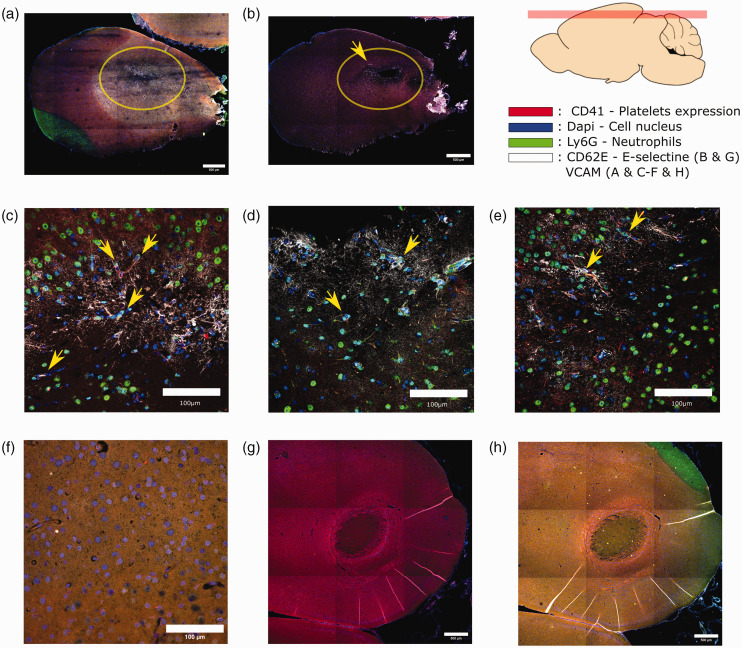
Immunofluorescence from 4 protein expressions is performed and shown to investigate the mechanism of the insults performed. DAPI was used to color the cell's nucleus in blue. The area of multiple capillary blockages is pointed out by a yellow circle where the labeling seems to be uniform throughout the region for a 4 hour brain post manipulations (a). Some 40× magnifications are shown (c–f). Yellow arrows highlight some points where expression of Ly6G(green), VCAM(white) and CD41(red) are visible and superimpose pointing towards a mechanism involving the neutrophil as a driver of the capillary blockage. CD41 expression tends to show disseminated platelets in the vessels which can be explained by stalled blood during the sacrifice. An example of healthy tissue is shown (f). Labeling on the image (b) shows little to no expression of CD41(red) and some expression of CD62E(white) in the morphologically changed area indicating inflammation at the vascular endothelium level. 24 hours after the insults(g and h), cicatricial tissue seems to appear in the center of the targeted region also pointing towards cellular death post oxygen deprivation. No expression of CD41(red (g and h)),CD62E(white (g)), Ly6G (green(h)) or VCAM(white(h)) are observed to be qualitatively different from healthy tissue.

An immunohistology investigation 4 hours and 24 hours following the insults was performed to elucidate the different processes occuring around the area of vascular blockage. The results shown in Figure 3(a) to (e) shows VCAM expression around the site where multiple capillaries were blocked (see Supp. Methods). Moreover, Ly6-G labeling shows a recruitment of monocytes/granulocytes/neutrophils in the center of the injured area. Some superpositions of Ly6G expression and VCAM/platelets are observable in the fixed tissue which are highlighted by yellow arrows. After 24 h, Figure 3(g) and (h) shows no presence of platelets, no E-selectin expression or neutrophils. However, appearance of cicatricial tissue and VCAM expression in some vessels can be observed in the area where insults were performed.

## Discussion

### Dye characterisation

We presented a novel way of manipulating the hemodynamic properties of targeted microvessels using a well established dye and off-the-shelf optical elements. We demonstrated the possibility of interrupting RBC flow in targeted vessels using a simple line scan protocol combined with multiphoton excitation of Rose Bengal. The characterized cross section at 1000 nm of this reagent (3 × 10^−60^ m^4^s/photons) is around 100 times lower than FitC's cross section (8 × 10^−58^ m^4^s/photons) for the same wavelength. Nonetheless, since FitC is a potent imaging dye in biological studies, a 100 fold difference in cross section is still usable in a well designed and optically isolated 2P microscopy setup as shown in our results. Our proposed methodology using a simple line scan protocol enabled blocking capillaries with a success rate of 60%, compared to 14% in a control group injected with FitC for the same optical power of ∼87 mW. Therefore, this change in occlusion rates and multiphoton response of the Rose Bengal are indicators that oxidative stress via ROS creation is likely the mechanism underlying the flow interruptions observed.

### RBC speed flux correlation and transient behavior

In contrast to the traditional green light illumination of Rose Bengal, our proposed multiphoton excitation of the photothrombotic agent does not seem to create permanent blockage of the targeted microvessels. In our experiments, we observed persistent blockages after the insult, 2 hours, 4 hours following a confirmed insult and a recovery of irrigation after 24 h (See supplementary video 2). Although more work is necessary to understand the root cause of this transient behavior, one candidate could be the RBC dynamics inside the vessel. With green light excitation, pial vessels are usually targeted to create larger clots via platelets activation and RBC assembly and ultimately blood flow cessation in downstream structures through lodging of these clots or local aggregation. Capillaries might not provide an environment as auspicious to the traditional blood clot formation mechanism since RBC flows discreetly in such vessels. Moreover, during the excitation of the Rose Bengal, correlation of RBC speed and flux seems to be conserved until the complete blockage. The inflammation caused by our protocol via ROS or heat deposition is not a tool we can use to disrupt the vessel enough to change the properties that constrain this correlation. Overall, the presented model might provide an avenue towards generating controlled insults via local inflammation creation.

### Insults mechanism

Through multiphoton excitation of Rose Bengal, we demonstrated here the possibility to perturb flow in a single microvessel optically. Nevertheless, the underlying mechanism linking the photostimulation remains to be investigated. In our trials, we observed that FitC excitation could cause vessel damages via hemorrhage, extravasation and blood flow cessation. Three hypotheses for these injury processes are FitC photo-saturation, laser damage via micro cavitation or heat deposition. To discard the micro cavitation hypothesis, it is important to consider the fluency of the laser at the imaging plane. Since the 2P source does not provide a fluency around the threshold of ablation of 1 J/cm^2^ in biological tissue,^18^ creation of a microcavitation bubble causing physical injury is implausible. For reference, computation of fluency with our setup provides a value around 0.1 J/cm^2^, 10× lower than the threshold level. With respect to the photo-saturation hypothesis, we suspect the saturation of the FitC dye plays a role as a phototoxicity reagent^28^ causing damage to the vascular walls similar to the mechanism seen with Rose Bengal. As Figure 1(f) and (g) suggests, the rate difference between the type of insults in Rose Bengal and FitC could be primarily explained by the efficacy of each probe to create reactive oxygen species. We cannot discard at this stage the contribution of heat deposition as a mechanism causing capillary injuries. Since blood flow cessation is more prominent in the Rose Bengal group, reactive oxygen species from this reagent and heat deposition are highly likely to play a main role in the insults created. Histological observations 4 hours after the injuries show a clear inflammatory response by the VCAM expression in tissue with an absence of agglomerates of platelets via the CD41 expression. Some CD41 expression is still observed, however this might be due to clogged vessels in the sacrificial process leaving disseminated platelets in the vessels. This observation tends to show that blocked vessels have experienced inflammatory processes without rupture or extravasation. As displayed in Figure 3, a superposition of Ly6G expression with VCAM/platelets can be observed in vessels expressing inflammatory damage. These spatially close expressions are evidence pointing towards a mechanism of neutrophil plugging as described in *Hernandez et al*. Moreover, E-selectin (CD62E) expression is observed in the vicinity of such insults, also demonstrating a trend towards an inflammation route at the vascular endothelium level. In the 24 hours post insults group, most infllamation seem to recede in the targeted area but limited VCAM expression is still present at region’s periphery. No other notable expressions of Ly6G, CD62E, CD41 are observed. However, the tissue seems to undergo a morphological change observable by DAPI labeling where fibrous structures seem to fill the area of insult which could be cicatricial tissue. This change in appearance can also be attributed to a rapid increase of ischemic cell deaths in the targeted area. Which would represent a penumbra in the cortical tissue caused by the multiple capillaries targeted in a roughly 1 mm square area. These observations and analysis point towards the activation of Rose Bengal and ROS creation as main drivers of inflammation leading to a blockage assisted by neutrophil adhesion at the vascular wall.

### Advantages of multiphoton photothrombosis

The primary result of this work is the excitation of Rose Bengal in a multiphoton setting. Although shifting to such a multiphoton scheme may reduce the efficiency of ROS creation, mainly due to the reduction in the probe's cross-section, it provides precious additional advantages. The targeting depth is increased in our configuration due to the increase in excitation wavelength, as near-infrared light is less susceptible to scattering in biological tissue. Additionally, the multiphoton process enables precise spatial targeting of vessels in the axial dimension, thus mitigating any residual illumination outside of the targeted area. Although protocols are being tailored to minimize secondary occlusions caused by scattering,^12^ single-photon excitation cannot provide the level of specificity demonstrated in our work. An intrinsic advantage from the multiphoton excitation of Rose Bengal comes from the fluorescence emitted during the line scans protocol ensuring precise localization of the photothrombosis effect. Our approach can be easily implemented in any multiphoton imaging system, and can therefore be complemented with other imaging dyes, enabling concurrent imaging of neuronal and oxygenation dynamics during the blockages.

### Limitations

This work presents the possibility of Rose Bengal usage for multiphoton targeted insults, but aspects concerning the characterization of the vascular topology and inflammation process remain obscure and are a limitation to the present study. No *in vitro* monitoring of the Rose Bengal ROS generated during 2P excitation was performed. We assumed that the same molecular pathways of the Rose Bengal molecule were activated in the creation of ROS. We suspect that multiphoton excitation may be less efficient regarding this aspect, but no result presented in this paper could prove such a claim. During our trials, we could not characterize the order of the vessel, but considered discrete RBC passage as a qualitative condition to identify the vessel as a capillary. Since the order of the vessel may influence the diffusion of generated ROS in the flowing blood and other hemodynamic parameters, this variable could have explained the high variance we observe in the time of excitation before blockage. Despite efforts to clarify the origin of OCMA signal, its relation to RBC flux and speed are unknown. Therefore, we used OCMA as a qualitative signal indicator of RBC passage. Since we cannot provide quantitative hemodynamics with OCMA, we could not corroborate RBC flux and speed values from our 2P acquired data. Additionally, the animals used in this study were all young mice, which can cause a bias over the observations. Since young mice are more susceptible to recover from such vascular insults as opposed to Alzheimer disease mice models or older mice. A supplementary limitation resides in the design of the FitC/Rose Bengal comparison groups. Since the FitC sessions were performed before the Rose Bengal group, we cannot completely discard the impact of the previous imaging session on the second. However, FitC being rapidly cleared from the system, the minimal waiting time of 3 days between imaging sessions and the fact that different cortical regions were used, we believe that residual inflammation that could have been caused by previous sessions were negligible.

### Future experiment

Possible uses of this technique could reside in the creation of subcortical insults near the white matter. Since clinical microinfarcts are observed throughout the cortical layers and the white matter in dementia, this technique could be a useful tool to perform photothrombosis near the cerebral white matter to corroborate observation of oxygen deprivation in these regions. Enhancing the attainable depth would be crucial for the method since depth is still a problematic factor in the time of excitation. Using sophisticated optical techniques, surgical manipulations or other photothrombotic agents with a multiphoton pathway could prove beneficial to increase the depth of targeted insults.

The limited duration of the insults (under 24 h) could prove beneficial in understanding and observing stalling events as well as recovery from nearby tissue. This model could provide a way to control stalling since such events are hard to study given their scarcity. With proper probes such as PO2 markers, analysis of the recovery of oxygen deprivation in controlled *in vivo* experiments and its consequences could help enhance our understanding of nutrients and oxygen diffusion at the capillary level.

A final application of this technique could exploit such control over the flow of one capillary branch combined with analysis of concurrent Doppler OCM^27^ to obtain a bigger picture from the impact of a single microvessel blockage and, at the same time, assessing the implications of peripheral vessels and changes in the vicinity of the target zone providing a framework for an *in vivo* corroboration of simulation *in silico* as presented in Schmid et al.^7^

## Conclusion

In this work, we demonstrate a novel way to use an off the shelf photothrombosis reagent via a multiphotonic activation pathway. Advantages of this technique rely on the longer wavelength used for the excitation, providing an opportunity for deeper cortical insults; the spatial confinement of the excitation volume which limit the activation of Rose Bengal and ensure a targeted single capillary occlusion and the disponibility of tools required that are ubiquitous to neuroscience labs. This protocol enables targeted inflammation driven capillary stalls as seen in neuropathologies characterized by neutrophils adhering to the vascular wall through VCAM/CD62E expression. Future work will focus on nutrient distribution and reach of influence of such insults.

## Supplemental Material

sj-pdf-1-jcb-10.1177_0271678X231151560 - Supplemental material for Targeted capillary photothrombosis via multiphoton excitation of Rose Bengal Click here for additional data file. Supplemental material, sj-pdf-1-jcb-10.1177_0271678X231151560 for Targeted capillary photothrombosis via multiphoton excitation of Rose Bengal by Patrick Delafontaine-Martel, Cong Zhang, Xuecong Lu, Rafat Damseh, Frédéric Lesage and Paul J Marchand in Journal of Cerebral Blood Flow & Metabolism

sj-pdf-2-jcb-10.1177_0271678X231151560 - Supplemental material for Targeted capillary photothrombosis via multiphoton excitation of Rose Bengal Click here for additional data file. Supplemental material, sj-pdf-2-jcb-10.1177_0271678X231151560 for Targeted capillary photothrombosis via multiphoton excitation of Rose Bengal by Patrick Delafontaine-Martel, Cong Zhang, Xuecong Lu, Rafat Damseh, Frédéric Lesage and Paul J Marchand in Journal of Cerebral Blood Flow & Metabolism

sj-jpg-3-jcb-10.1177_0271678X231151560 - Supplemental material for Targeted capillary photothrombosis via multiphoton excitation of Rose Bengal Click here for additional data file. Supplemental material, sj-jpg-3-jcb-10.1177_0271678X231151560 for Targeted capillary photothrombosis via multiphoton excitation of Rose Bengal by Patrick Delafontaine-Martel, Cong Zhang, Xuecong Lu, Rafat Damseh, Frédéric Lesage and Paul J Marchand in Journal of Cerebral Blood Flow & Metabolism

sj-jpg-4-jcb-10.1177_0271678X231151560 - Supplemental material for Targeted capillary photothrombosis via multiphoton excitation of Rose Bengal Click here for additional data file. Supplemental material, sj-jpg-4-jcb-10.1177_0271678X231151560 for Targeted capillary photothrombosis via multiphoton excitation of Rose Bengal by Patrick Delafontaine-Martel, Cong Zhang, Xuecong Lu, Rafat Damseh, Frédéric Lesage and Paul J Marchand in Journal of Cerebral Blood Flow & Metabolism

## References

[1] Cruz Hernández JC Bracko O Kersbergen CJ , et al. Neutrophil adhesion in brain capillaries reduces cortical blood flow and impairs memory function in Alzheimer’s disease mouse models. Nat Neurosci 2019; 22: 413–420. 30742116 10.1038/s41593-018-0329-4 PMC6508667

[2] Erdener SE Tang J Kılıç K , et al. A hyperacute role for neutrophils in persistent traffic jams. J Cereb Blood Flow Metab 2021; 41: 236–252. 32237951 10.1177/0271678X20914179 PMC8370003

[3] Lu Y Lu X Zhang C , et al. Longitudinal optical coherence tomography imaging of tissue repair and microvasculature regeneration and function after targeted cerebral ischemia. J Biomed Opt 2020; 25: 1–15. 10.1117/1.JBO.25.4.046002 PMC7152803 32285652

[4] Erdener SE Tang J Sajjadi A , et al. Spatio-temporal dynamics of cerebral capillary segments with stalling red blood cells. J Cereb Blood Flow Metab 2019; 39: 886–900. 29168661 10.1177/0271678X17743877 PMC6501506

[5] Hartung G Badr S Moeini M , et al. Voxelized simulation of cerebral oxygen perfusion elucidates hypoxia in aged mouse cortex. PLOS Comput Biol 2021; 17: e1008584. 33507970 10.1371/journal.pcbi.1008584 PMC7842915

[6] Lu X Moeini M Li B , et al. Voluntary exercise increases brain tissue oxygenation and spatially homogenizes oxygen delivery in a mouse model of Alzheimer's disease. Neurobiol Aging 2020; 88: 11–23. 31866158 10.1016/j.neurobiolaging.2019.11.015

[7] Schmid F Conti G Jenny P , et al. The severity of microstrokes depends on local vascular topology and baseline perfusion. Elife 2021; 10: e60208. 34003107 10.7554/eLife.60208 PMC8421069

[8] Watson BD Dietrich WD Busto R , et al. Induction of reproducible brain infarction by photochemically initiated thrombosis. Ann Neurol 1985; 17: 497–504. 4004172 10.1002/ana.410170513

[9] Shih AY Blinder P Tsai PS , et al. The smallest stroke: occlusion of one penetrating vessel leads to infarction and a cognitive deficit. Nat Neurosci 2013; 16: 55–63. 23242312 10.1038/nn.3278 PMC3952571

[10] Schaffer CB Friedman B Nishimura N , et al. Two-photon imaging of cortical surface microvessels reveals a robust redistribution in blood flow after vascular occlusion. PLoS Biol 2006; 4: 258–270. 10.1371/journal.pbio.0040022 PMC1324794 16379497

[11] Shams Kazmi SM Salvaggio AJ Estrada AD , et al. Three-dimensional mapping of oxygen tension in cortical arterioles before and after occlusion. Biomed Opt Express 2013; 4: 1061–1073. 23847732 10.1364/BOE.4.001061 PMC3704088

[12] Sunil S Erdener SE Lee BS , et al. Awake chronic mouse model of targeted pial vessel occlusion via photothrombosis. Neurophoton 2020; 7: 015005. 10.1117/1.NPh.7.1.015005 PMC6992450 32042854

[13] Svoboda K Ryohei Y. Principles of two-photon excitation microscopy and its applications to neuroscience. Neuron 2006; 50: 823–839. 16772166 10.1016/j.neuron.2006.05.019

[14] Mittmann W Wallace DJ Czubayko U , et al. Two-photon calcium imaging of evoked activity from L5 somatosensory neurons in vivo. Nat Neurosci 2011; 14: 1089–1093. 21743473 10.1038/nn.2879

[15] Carrillo-Reid L Han S Yang W , et al. Controlling visually guided behavior by holographic recalling of cortical ensembles. Cell 2019; 178: 447–457. 31257030 10.1016/j.cell.2019.05.045 PMC6747687

[16] Shih AY Driscoll JD Drew PJ , et al. Two-photon microscopy as a tool to study blood flow and neurovascular coupling in the rodent brain. J Cereb Blood Flow Metab 2012; 32: 1277–1309. 22293983 10.1038/jcbfm.2011.196 PMC3390800

[17] Sakadžić S Roussakis E Yaseen M , et al. Two-photon high-resolution measurement of partial pressure of oxygen in cerebral vasculature and tissue. Nat Methods 2010; 7: 755–759. 20693997 10.1038/nmeth.1490 PMC2932799

[18] Nishimura N Schaffer CB Friedman B , et al. Targeted insult to subsurface cortical blood vessels using ultrashort laser pulses: three models of stroke. Nat Methods 2006; 3: 99–108. 16432519 10.1038/nmeth844

[19] Srinivasan VJ Radhakrishnan H Jiang JY , et al. Optical coherence microscopy for deep tissue imaging of the cerebral cortex with intrinsic contrast. Opt Express 2012; 20: 2220–2239. 22330462 10.1364/OE.20.002220 PMC3306182

[20] Lu X Moeini M Li B , et al. Hypertension accelerates cerebral tissue PO2 disruption in Alzheimer’s disease. Neurosci Lett 2020; 715: 134626. 31726177 10.1016/j.neulet.2019.134626

[21] Drew PJ Blinder P Cauwenberghs G , et al. Rapid determination of particle velocity from space-time images using the radon transform. J Comput Neurosci 2010; 29: 5–11. 19459038 10.1007/s10827-009-0159-1 PMC4962871

[22] Münch B Trtik P Marone F , et al. Stripe and ring artifact removal with combined wavelet – Fourier filtering. Opt Express 2009; 17: 8567–8591. 19434191 10.1364/oe.17.008567

[23] Sato Y Nakajima S Shiraga N , et al. Three-dimensional multi-scale line filter for segmentation and visualization of curvilinear structures in medical images. Med Image Anal 1998; 2: 143–168. 10646760 10.1016/s1361-8415(98)80009-1

[25] Li CH Lee CK. Minimum cross entropy thresholding. Pattern Recognition 1993; 26: 617–625.

[24] Gilles J-F Dos Santos M Boudier T , et al. DiAna, an ImageJ tool for object-based 3D co-localization and distance analysis. Methods 2017; 115: 55–64. 27890650 10.1016/j.ymeth.2016.11.016

[26] Xu C Webb WW. Measurement of two-photon excitation cross sections of molecular fluorophores with data from 690 to 1050 nm. J Opt Soc Am B 1996; 13: 481–491.

[27] Srinivasan VJ Yu E Radhakrishnan H , et al. Micro-heterogeneity of flow in a mouse model of chronic cerebral hypoperfusion revealed by longitudinal doppler optical coherence tomography and angiography. J Cereb Blood Flow Metab 2015; 35: 1552–1560. 26243708 10.1038/jcbfm.2015.175 PMC4640323

[28] Rumbaut RE Sial AJ. Differential phototoxicity of fluorescent dye-labeled albumin conjugates. Microcirculation 1999; 6: 205–213. 10501094

