# Supplementary material for: Targeted capillary photothrombosis via multiphoton excitation of Rose Bengal
Source: J Cereb Blood Flow Metab. 2023 Jan 17;43(10):1713–25. doi: 10.1177/0271678X231151560 (PMC10581236; doi:10.1177/0271678X231151560)
Supplement: sj-pdf-1-jcb-10.1177_0271678X231151560 - Supplemental material for Targeted capillary photothrombosis via multiphoton excitation of Rose Bengal [file sj-pdf-1-jcb-10.1177_0271678X231151560.pdf]

Supplementary Methods

Optical system

A multimodal platform was designed for simultaneous Optical Coherence Microscopy (OCM) and 2P *in vivo* imaging (shown in Fig. 1a). The OCM system consists of a light source operating at 1300 nm (LS2000C, Thorlabs), split into the sample and reference arm using a 90/10 fiber beam splitter, after crossing through a fiberized circulator. Two pre-aligned delay lines were available and selected via a mirror fitted flip stage (Thorlabs) for easy delay-line switch between a 10X Mitutoyo Plan Apo and a Olympus XLPLN25XWMP2 OCM objectives. The dispersion was balanced by adding glass prisms (SF10 and UVFS) in the reference arm. Residual dispersion was adjusted numerically. The 2P microscope is composed of a femtosecond laser (MaiTai, Spectra-Physics), a Pockels cell (ConOptics) and PMTs (H7422-50, Hamamatsu Photonics, Japan). The detection and excitation paths were separated before the objective using a long-pass dichroic mirror (Semrock). 2P imaging was performed at 920 nm and 1000 nm for the FitC and Rose Bengal respectively. Fluorescence was collected through emission filters centered at 520 nm and 580 nm (Semrock). All 2P imaging sessions used the Olympus objective and the OCM sessions used either objective. A commercial spectrometer was employed to register interferograms, based on a high-speed 2048 pixels InGaAs line camera (Wasatch Photonics). Light from the OCM path was merged with the 2P path using a short-pass dichroic mirror (Semrock) and then directed to the microscope's objective. A single computer controlled both platforms, but separate acquisition boards (National Instruments) were used for each modality.

Optical coherence microscopy angiography

Observing the irrigation status of insulted vessels using the protocol devised here entailed imaging Rose Bengal filled vessels using the multiphoton excitation setup, potentially activating the photothrombotic agent leading to undesired vessel blockage. To alleviate this problem, we performed simultaneous angiography imaging using OCM, an interferometric label-free imaging technique[19]. Demonstration of the aforementioned technique as an efficient monitoring method was shown through observation of stalls in a high throughput *in vivo* setup[20] and in an impaired microvascular system via photothrombosis caused by conventional green light excitation of Rose Bengal[3]. Moreover, we employed an extension of OCM, termed OCM Angiography (OCMA) enabling selective visualization of perfused regions in the cortex, through a high frequency temporal filtering differentiating between dynamic RBCs and static tissue. As OCMA relies on a low power near infrared light source, ideal to circumvent the agent activation problem. Overall, after consideration of the stalling monitoring capacity and the lack of ROS creation with the hemodynamic monitoring, we chose concurrent OCMA imaging to verify irrigation status.

Timetraces processing

Quantification of RBC speed and flux from the linescans was performed using a framework inspired by Drew et al.[22] Line scans were sorted out in 512*512 images (time x space) which were used to perform Radon transforms (skimage.transform.radon in Python) and find the preferential angle of RBCs. Speed was computed from the frequency of acquisition and the spatial length of the scan. Subsequently, rotation of the data was applied to create an image containing perpendicular RBC shadows. This manipulation provided a sharper mean projection of the

resulting image corresponding to the RBC profile. These monitoring data were then passed to a one dimensional median filter with a 5 pixel kernel to remove speckle noise. Scipy's find peaks function (scipy.signal.find_peaks) was then applied to identify each RBC. The RBC flux was computed via the number of peaks found in the image divided by the time associated with the 512 pixels acquisition.

OCT image processing and analysis

To enhance the quality of the OCMA acquisitions, various steps were used. A 3D median filter with a kernel of 3*3*3 pixels is first performed to remove any salt and pepper artifacts in the images. Line artifacts caused by movements were removed in MATLAB via the combined wavelet and Fourier filtering algorithm presented by Münch et al[23]. Sato filtering[24] was then performed to amplify tube-like structures in the data followed by an automatic Li threshold technique[25]. To recover distances from the nearest vessel in OCMA images, a toolkit in the ImageJ platform called Distance 3D transform[26] was utilized. 2P scans were treated similarly with the exception of the line artifact removal. Most of the analysis was implemented in a Python environment using Sci-Kit image and Scipy libraries.

Statistical analysis

A binary z-test was coded in Python using the proportions z test function in the statsmodels.stats.proportion module with default settings. Such a test was provided with an input of 0 and 1 representing the unchanged flow of the vessel or the observed blockage respectively. We ensured a large population dataset (n>100) which is represented by the trial of photothrombosis for the control group and the experimental group (see below).

Histological manipulation and labeling

At sacrifice, brains were perfused and fixed in a 4% PFA solution for 24h. The samples were conserved in a 1x PBS solution for up to 2 weeks. A dehydration method using 6 ethanol concentrations was performed, 1 hour in each (70%, 95%, 95%, 100%, 100%, 100%). The 3 last immersions were performed under vacuum. Immersions in xylene and paraffin were then done. The embedded brain was then cut in a microtome at a 6um thickness and samples placed on a microscope glass slide to undergo histological labeling. Four markers were selected to detect inflammation or neutrophil presence : VCAM, CD41, CD62E, Ly6G. A citrate buffer solution immersion of the slides was done prior to the immunostaining. Immersion in a monoclonal anti-mouse Ly6G -1A8 - Alexa 488 (Rat) solution was done at concentration 25ug/ml for neutrophil labeling. For the other markers, secondary antigen labeled with Alexa fluorochromes were used with a concentration of 5 ug/ml after the immersion in their primary antigen counterpart following a rinse in the host serum between the primary and secondary antigen solution. To ensure multiple labeling per slide, different hosts were selected (Donkey anti-goat anti-VCAM1, Rat anti-rabbit anti-CD41, Rabbit Anti-Rat anti-CD62E). This combination of antigens excluded the labeling of both VCAM and CD62E on the same slide. Hence, consecutive slides were labeled alternatively between these two markers.
