# Supplementary material for: Targeted capillary photothrombosis via multiphoton excitation of Rose Bengal
Source: J Cereb Blood Flow Metab. 2023 Jan 17;43(10):1713–25. doi: 10.1177/0271678X231151560 (PMC10581236; doi:10.1177/0271678X231151560)
Supplement: sj-pdf-2-jcb-10.1177_0271678X231151560 - Supplemental material for Targeted capillary photothrombosis via multiphoton excitation of Rose Bengal [file sj-pdf-2-jcb-10.1177_0271678X231151560.pdf]

Supplementary Material Legends:

Supplementary Figure 1 : A timeline representation of the methodology used in this experiment is presented. This figure is a recapitulative view of what is explained in the paragraph titled ‘Animal groups’ (M : Males, F : Females). 2 trials were used, 1 for the overall analysis and a second trial to focus on the blockage monitoring.

Supplementary Figure 2 : A collection of line scans is presented. Speed and RBC flux are compared to highlight the correlation between both measurements. All blockages presented here were certified to be blocked with a second imaging up to 5 minutes later. Different blockages mechanics are observable from sudden blockage to long descending RBC flux.

Supplementary videos 1 : Two photon microscopy imaging linescans data are shown. The signal is from the Rose Bengal in the plasma. One can see the RBC flux until the cessation of flow. The y-axis represents the time, the x-axis is the travel of the point of laser excitation. The changing image also represents times as a stack. The acquisition parameters are provided in the methodology.

Supplementary video 2 : Video presenting OCMA data before the insult. Flow can be seen from the targeted vessel before the 2P excitation.

Supplementary video 3 : Video presenting OCMA data after the insult. Flow has been interrupted after the Rose bengal excitation.

Supplementary video 4 : Video presenting OCMA data 4 hours after the insult. Flow is interrupted constantly for up to 4 hours.

Supplementary video 5 : Video presenting OCMA data 24 hours after the insult. Blood flow in the capillary recovers in most cases.

Supplementary Material :

Supplementary Figure 1 Here

Supplementary Figure 2 Here

Supplementary video 1 Here

Supplementary video 2 Here

Supplementary video 3 Here

Supplementary video 4 Here

Supplementary video 5 Here
