# Supplementary figures and images for: Targeted capillary photothrombosis via multiphoton excitation of Rose Bengal
Source: J Cereb Blood Flow Metab. 2023 Jan 17;43(10):1713–25. doi: 10.1177/0271678X231151560 (PMC10581236; doi:10.1177/0271678X231151560)

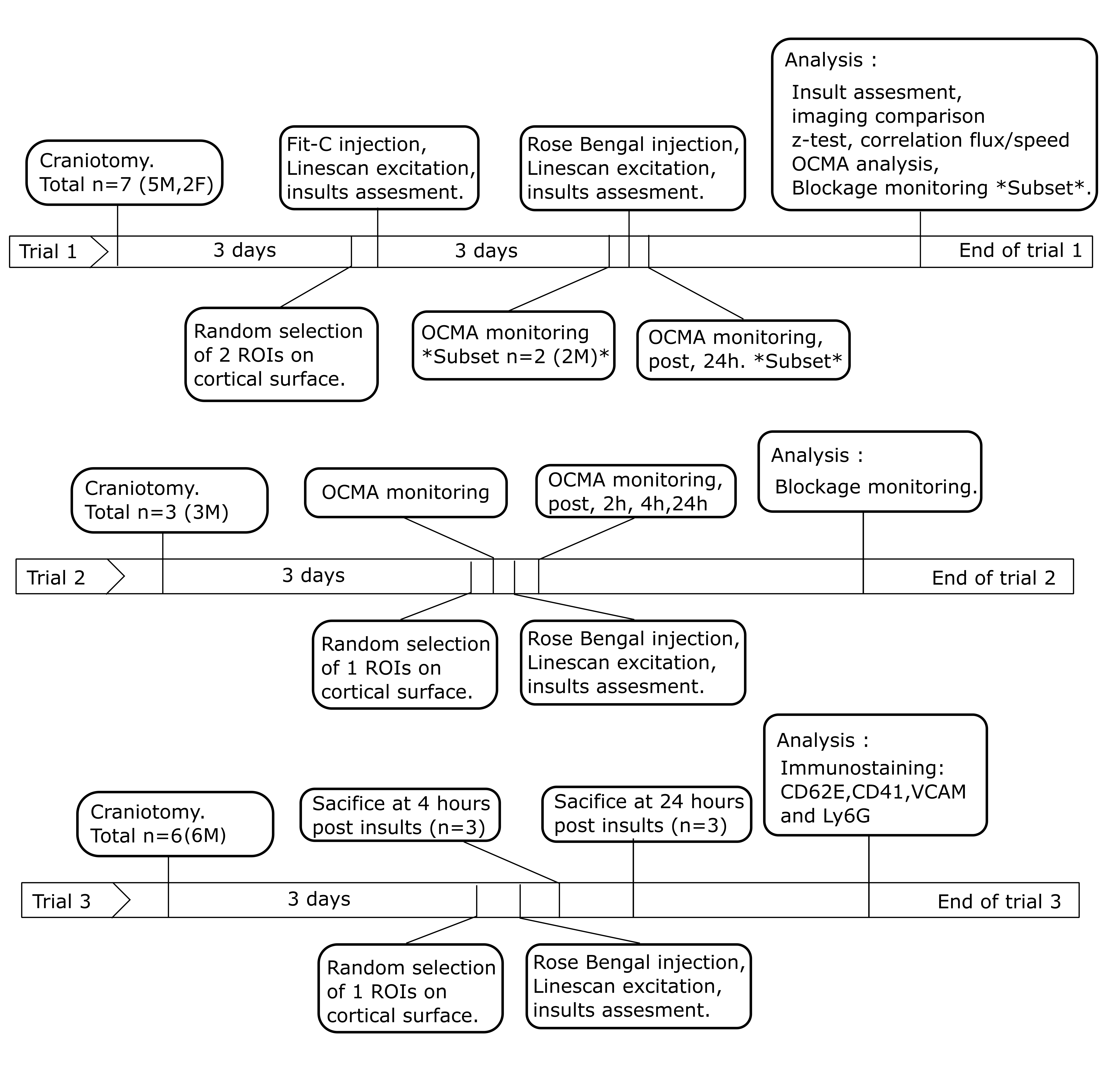

Supplement: sj-jpg-3-jcb-10.1177_0271678X231151560 - Supplemental material for Targeted capillary photothrombosis via multiphoton excitation of Rose Bengal [file sj-jpg-3-jcb-10.1177_0271678X231151560.jpg]
